# Experimental Study of the Biological Properties of Human Embryonic Stem Cell–Derived Retinal Progenitor Cells

**DOI:** 10.1038/srep42363

**Published:** 2017-02-13

**Authors:** Jingzhi Shao, Peng-Yi Zhou, Guang-Hua Peng

**Affiliations:** 1 Department of Ophthalmology, The First Affiliated Hospital of Zhengzhou University, Zhengzhou, Henan 450000, China; 2 Department of Ophthalmology, General Hospital of Chinese People’s Liberation Army, Beijing 100853, China

## Abstract

Retinal degenerative diseases are among the leading causes of blindness worldwide, and cell replacement is considered as a promising therapeutic. However, the resources of seed cells are scarce. To further explore this type of therapy, we adopted a culture system that could harvest a substantial quantity of retinal progenitor cells (RPCs) from human embryonic stem cells (hESCs) within a relatively short period of time. Furthermore, we transplanted these RPCs into the subretinal spaces of Royal College of Surgeons (RCS) rats. We quantified the thickness of the treated rats’ outer nuclear layers (ONLs) and explored the visual function via electroretinography (ERG). It was found that the differentiated cells expressed RPC markers and photoreceptor progenitor markers. The transplanted RPCs survived for at least 12 weeks, resulting in beneficial effects on the morphology of the host retina, and led to a significant improvement in the visual function of the treated animals. These therapeutic effects suggest that the hESCs-derived RPCs could delay degeneration of the retina and partially restore visual function.

Retinal degeneration, such as age-related macular degeneration and retinitis pigmentosa, is initiated by the retinal pigment epithelium (RPE) cells and photoreceptor cells^1^,^2^. The mammalian eyes cannot regenerate photoreceptors and RPE cells^3^, and therefore, cell replacement, visual prosthetics, gene therapy, and drug therapy are most frequently used strategy to deal with this type of diseases.

Cell replacement has been proven to be the most feasible and promising method of treating retinal degeneration because specific cells transplanted into the subretinal space can integrate into the host retina and restore some retinal function^4^. MacLaren^5^ showed that the transplanted postmitotic photoreceptor precursor cells (PPCs) could integrate with the host retina and establish synaptic connections with interneurons. Furthermore, several studies have shown that the RPCs transplanted into retinal degenerative animal models could migrate into the outer retina and differentiate into photoreceptor cells. However, the sources of postmitotic PPCs and human progenitor cells (HPCs) are extremely scarce. Consequently, the most urgent problem is to obtain enough immature postmitotic PPCs and human RPCs to implement the therapeutic strategy. In the present study, we used immature postmitotic PPCs and HPCs as the sources of retinal progenitor cells (RPCs).

The ESCs, which can self-renew and differentiate into any other type of cell, are the most promising sources of PPCs and RPCs. It has been shown that embryonic stem cells (ESCs), Muller cells, mesenchymal stem cells, and some other cells can be induced to develop into RPCs or photoreceptor cells^6^,^7^,^8^,^9^,^10^. Several studies have developed successfully the protocols to induce ESCs or RPCs to differentiate into photoreceptors^11^,^12^,^13^,^14^. However, it is crucial to find an efficient method of harvesting the PPCs and RPCs in relative large quantities within a short period of time. Therefore, the aim of the present study was to develop an effective culture protocol. To do this, we transplanted the hESCs-derived RPCs into the subretinal spaces of 3-week-old RCS rats, which have served as the classic animal models of retinal degeneration involving the progressive apoptosis of photoreceptor cells^15^. Subsequently, we examined the histological structure and visual function of the treated rats, and found that the transplanted RPCs survived for at least 12 weeks, resulting in beneficial effects on the morphology of outer nuclear layer (ONL), and leading to significant improvement in the treated animals’ visual function. These therapeutic effects suggest that the hESCs-derived RPCs can delay degeneration of the retina and partially restore visual function without any adverse effects.

## Results

### Declining Ability of hESCs to Proliferate

**Figure 1:**
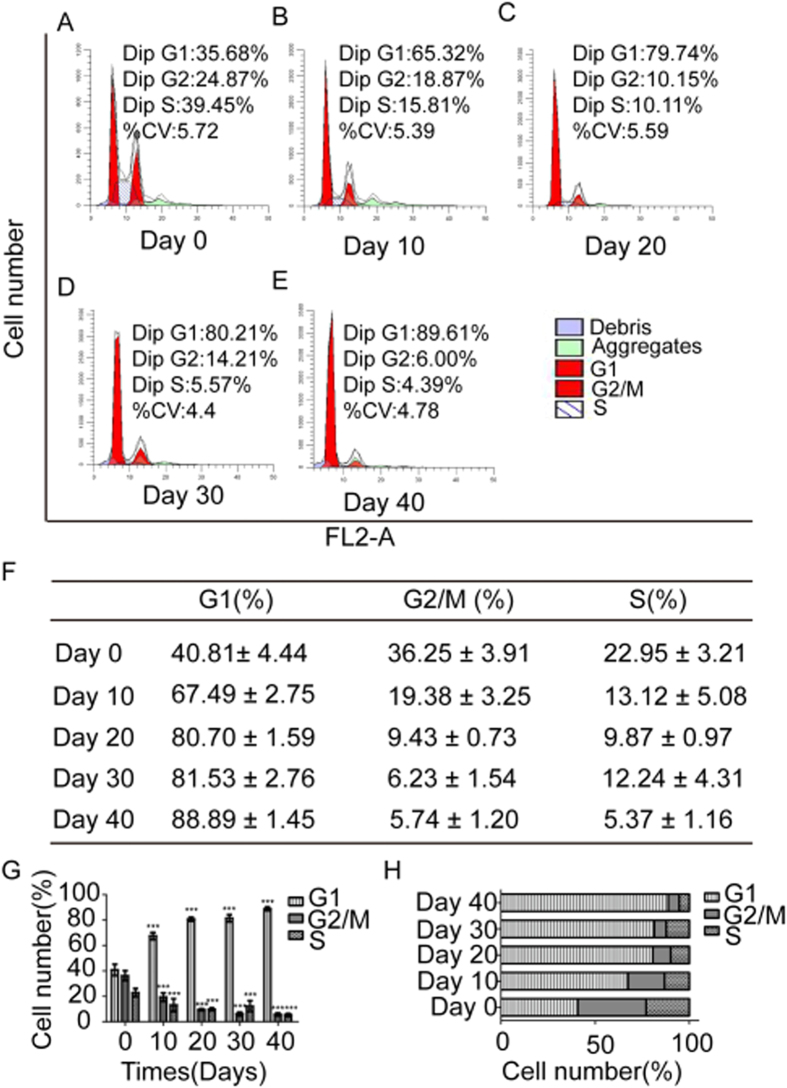
Cell cycle analysis of differentiated cells at 0, 10, 20, 30, and 40 days. (**A** to **E**) Cell cycle showed that the mitotic ratio was highest at day 0 of differentiation and then decreased. (**F**) Data of cell cycle at different times were shown as mean ± standard deviation (SD) in the form of table. (**G** to **H**) Statistical analysis showed the mitotic ratio of cells at different times. Data from at least three independent experiments are represented as the mean ± SD. All data expressed as mean ± SD. ***P < 0.001 versus with data of day 0.

We examined the hESC cell cycle of differentiating cells at different time points. Results showed that the percentages of cells in particular phases of cell cycle were 40.81 ± 4.44%, 36.25 ± 3.91%, and 22.95 ± 3.21% respectively, and the mitotic ratio was significantly highest on the 0th day, then it decreased with time passing (*P* < 0.001) (Fig. 1).

The percentage of Ki67-positive cells were 86.40 ± 5.54%, 44.23 ± 3.29%, 15.10 ± 4.32%, and 7.87 ± 1.69% respectively on the 0th, 10th, 20th, 30th, 40th days as detected by flow cytometry. Differentiation was statistically significant between the examined time point and the previous time point (*P* < 0.05) (Fig. S1B,C). These findings agreed well with the results of immunofluorescence (Fig. S1A).

### Expressions of Retinal Progenitor Cell Markers examined by Flow Cytometry and Immunofluorescence

**Figure 2:**
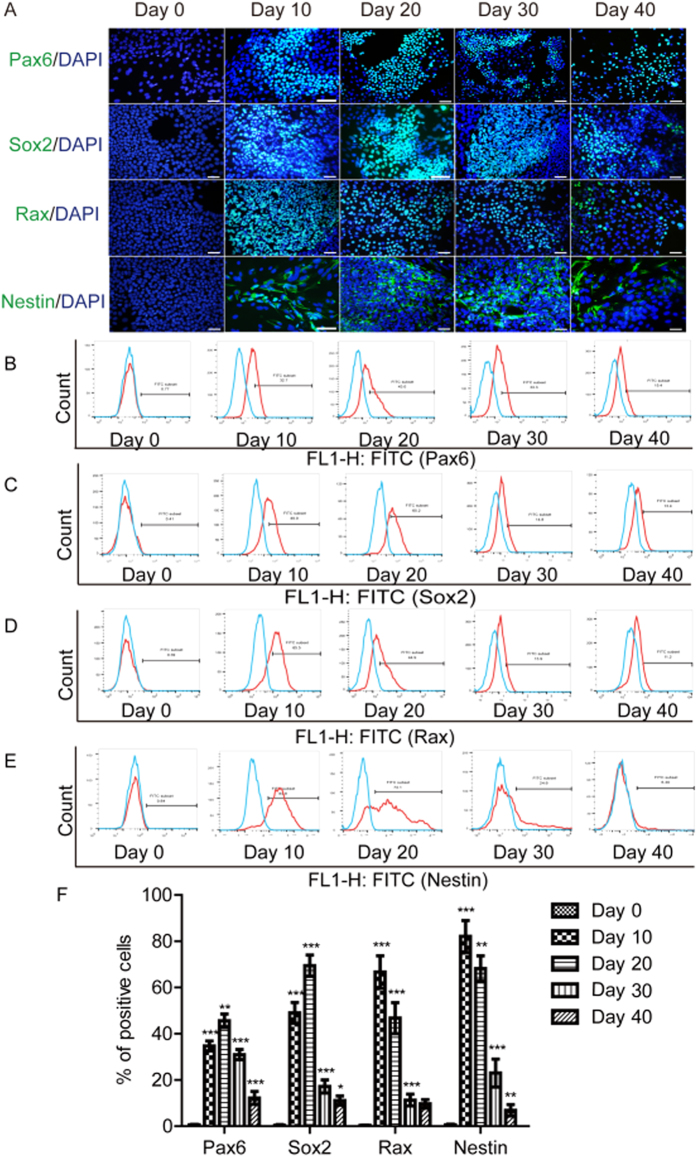
hESCs differentiated toward a RPC phenotype. (**A**) H1 differentiated into a RPC and expressed cell-specific markers—Pax6, Sox2, Rax, and Nestin, evaluated by Immunofluorescence. (**B** to **E**) Staining of Pax6, Sox2, Rax, and Nestin was evaluated by using FACS. Blue line, isotype. (**F**) Statistical analysis of expression of Pax6, Sox2, Rax, and Nestin. Cell nuclei are shown in 6-diamidino-2-phenylindole (DAPI), blue. Data from at least three independent experiments are represented as the mean ± SD. Scale bars = 25 μm. *P < 0.05, **P < 0.01, ***P < 0.001, versus with data of previous time point.

In order to induce the H1 to differentiate toward a retinal fate, we used a modified protocol following the previously described method^16^. We tested the efficiency of retinal determination by analyzing the expressions of several key transcription factors (including *Pax6, Sox2, Rax*, and *Nestin*) of the eyes using FACS and immunofluorescence (Fig. 2) and analyzed the time course of protein expressions (Fig. 2). On the 0th, 10th, 20th, 30th, 40^th^ days of differentiation, the percentages of Pax6-positive cell numbers were 0.63 ± 0.16%, 34.73 ± 2.10%, 45.63 ± 2.94%, 30.97 ± 2.21%, and 12.23 ± 2.79% respectively; the percentages of Sox2-positive cell numbers were 0.40 ± 0.27%, 49.07 ± 4.51%, 69.47 ± 4.58%, 17.20 ± 2.82%, and 11.10 ± 1.99% respectively; the percentages of Rax-positive cell numbers were 0.31 ± 0.18%, 66.60 ± 6.98%, 46.77 ± 6.69%, 11.33 ± 2.50%, and 9.70 ± 1.87% respectively; the percentages rates of Nestin-positive cell numbers were 0.61 ± 0.27%, 82.10 ± 6.86%, 68.20 ± 5.47%, 23.00 ± 6.06%, and 6.90 ± 2.46% respectively. The percentages of Pax6 and Sox2 positive cell numbers crested at the 20th day and were significantly higher compared with the percentages at the 10th day (*P* < 0.001). They then gradually declined until the 40th day and the differences were statistically significant compared with that at the 30th day (*P* < 0.01). The percentages of Rax and Nestin positive cell numbers crested at the 20th day and then gradually declined until the 40th day (*P* < 0.05).

**Figure 3:**
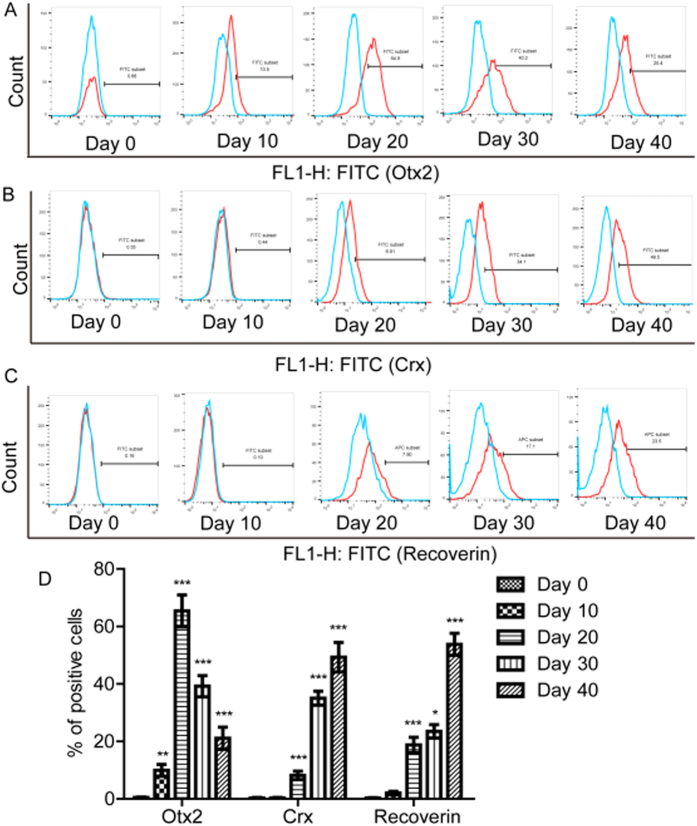
hESCs differentiated toward a PPC phenotype. (**A** to **C**) H1 differentiated into a PPC and expressed cell-specific markers—Otx2, Crx, and Recoverin, examined by FACS. (**D**) Statistical analysis of expression of Otx2, Crx, and Recoverin. Data from at least three independent experiments are represented as the mean ± SD. *P < 0.05, **P < 0.01, ***P < 0.001, versus with data of previous time point.

Moreover, we examined the expression levels of PPC and photoreceptor markers Otx2, Crx, and Recoverin using the FACS. On the 0th, 10th, 20th, 30th, 40th days of differentiation, the rates of Otx2-positive cell numbers were 0.49 ± 0.16%, 9.93 ± 2.01%, 65.40 ± 5.57%, 39.20 ± 3.70%, and 21.10 ± 3.89% respectively (Fig. 3A); the rates of Crx-positive cell numbers were 0.30 ± 0.17%, 0.31 ± 0.19%, 8.17 ± 1.48%, 35.07 ± 2.40%, and 49.30 ± 5.10% respectively (Fig. 3B); the rates of Recoverin-positive cell numbers were 0.24 ± 0.18%, 0.72 ± 0.55%, 6.77 ± 0.95%, 16.49 ± 1.99%, and 24.14 ± 3.04% respectively (Fig. 3C). We also analyzed the expression levels of the hESC marker SSEA-4, which stood at 98.80 ± 1.02% on the 0th day and then declined sharply. The rates of SSEA-4-positive cell numbers at the 10th, 20th, 30th, 40th days were 25.47 ± 2.83%, 7.64 ± 1.13%, 2.21 ± 0.79% and 0.93 ± 0.44% respectively (Fig. S2).

### Expressions of Retinal Progenitor Cell Markers Examined by Western Blot and Real-Time Polymerase Chain Reaction (Real-Time PCR)

**Figure 4:**
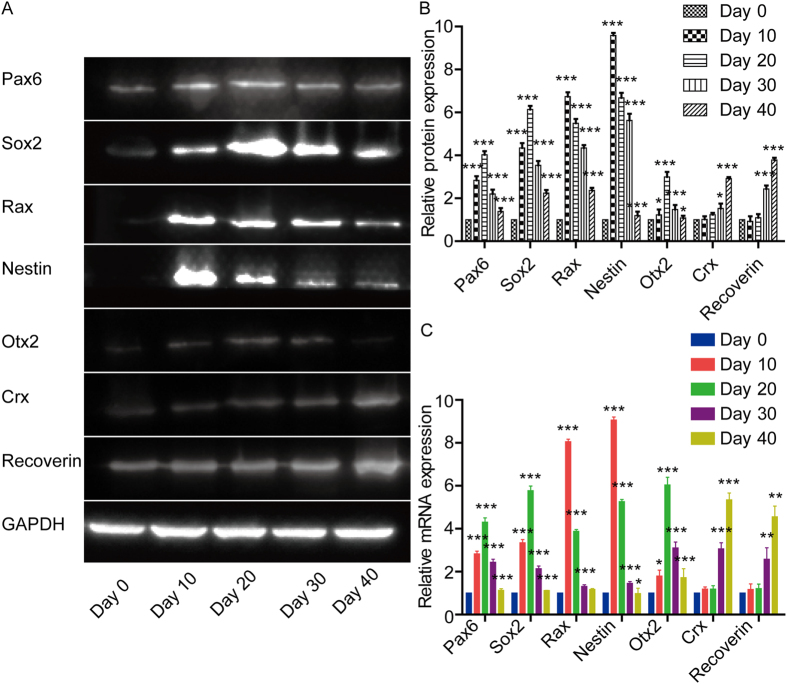
The expression level of proteins and genes of retinal progenitor cells during the differentiated development of hESCs analyzed by Western blot and real-time PCR. (**A**) Western blot analysis of retinal progenitor cell markers (Pax6, Sox2, Rax, Nestin, Otx2, Crx, and Recoverin) during their differentiated development. The gels in this experiment were run under the same experimental conditions. (**B**) Statistical analysis of the expression levels of proteins in retinal progenitor cell markers (Pax6, Sox2, Rax, Nestin, Otx2, Crx, and Recoverin). Data expressed as mean ± SD. (**C**) Statistical analysis of the expression of mRNA in retinal progenitor cell markers (*Pax6, Sox2, Rax, Nestin, Otx2, Crx, and Recoverin*). Data from at least three independent experiments are represented as the mean ± SD. *P < 0.05, **P < 0.01, ***P < 0.001, versus with data of previous time point.

We examined the protein expressions of Pax6, Sox2, Rax, Nestin, Otx2, Crx, and Recoverin on the 0, 10th, 20th, 30th, 40th days of differentiation by Western blot and real-time PCR. At the 10th day, the expressions of retinal progenitor cell markers (Rax and Nestin) reached crest, and then decreased over time. The expressions of retinal progenitor cell markers Pax6 and Rax increased over time, reached top at the 20th day, and then decreased over time. The expressions of photoreceptor precursor cell marker Otx2 reached its crest at 20th day. In contrast, Crx and Recoverin, which were also markers of photoreceptor cells progressively increased over time (Fig. 4).

### RPCs Survived, Integrated, and Delayed Degeneration of the Retina After Being Injected Into the Subretinal Spaces of the RCS Rats

**Figure 5:**
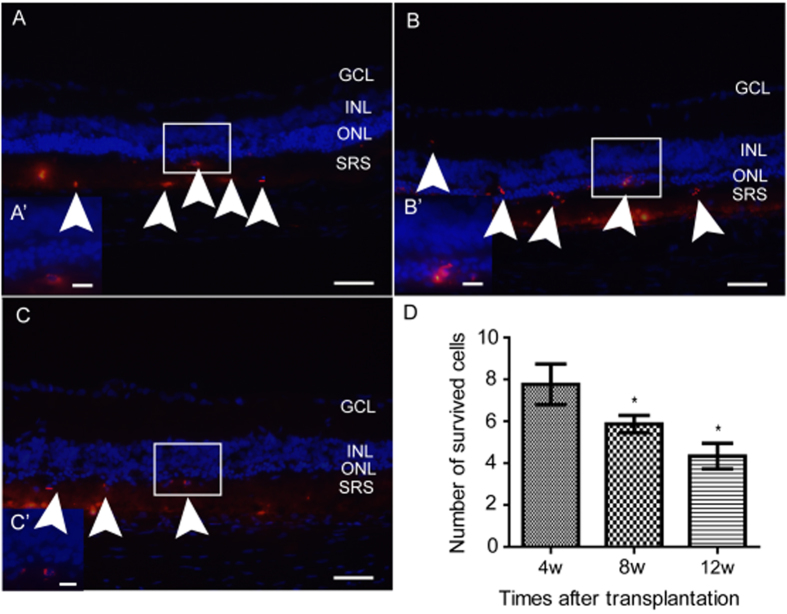
CM-Dil–labeled transplanted cells (red) survived in the host retina and migrated into the ONL. Micrographs represented the superior region of the retina. (**A** to **C**) showed the surviving cells in the images at 4 weeks (A), 8 weeks (**B**), and 12 weeks (**C**) after transplantation. (A’ to C’) magnified images of the small white rectangles in the centers showing cells integrated into the ONL. (**D**) The number of surviving cells in a section decreased as the time since transplantation grew longer. Data from at least three independent experiments are represented as the mean ± SD. *P < 0.05, versus with data of previous time point. Cell nuclei are shown in blue with DAPI; transplanted cells are shown in red; white arrowheads indicate surviving cells in the ONL and subretinal space. GCL, ganglion cells layer; INL, inner nuclear layer; ONL, outer nuclear layer; SRS, subretinal space. A to C scale bars = 100 μm; A’ to C’ scale bars = 25 μm.

We transplanted the hESC-derived RPCs (excluded SSEA-4^+^ cells by FACS) into the subretinal spaces of 21-day-old RCS rats with a surgical microscope after staining with Cell Tracker CM-Dil. Rats were sacrificed 4, 8, and 12 weeks after subretinal injection. It was found that the CM-Dil–labeled cells migrated from the subretinal spaces to the ONL with robust vitality (Fig. 5). Gradually, the number of surviving cells in the host retina gradually decreased over time (respectively 8.0 ± 0.27 cells, 6.75 ± 0.37 cells, and 4.5 ± 0.33 cells in a microscopic field with 400× magnification at the 4th, 8th, and 12th week (Fig. 5D).

**Figure 6:**
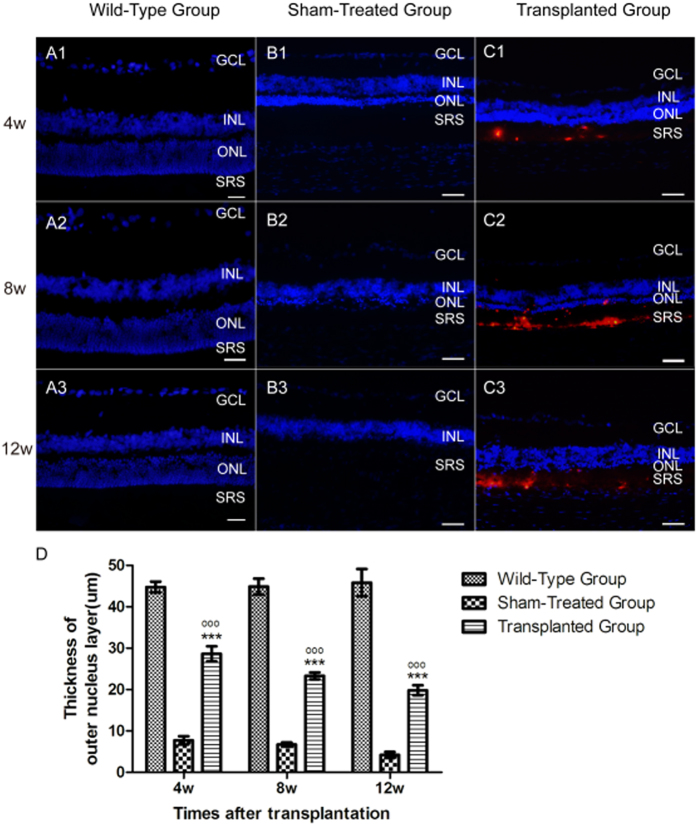
The micrographs showing the thicknesses of the ONL across the vertical meridian. (A1 to A3) Retinas of Long-Even rats at 4 weeks, 8 weeks, and 12 weeks. The average ONL thicknesses were 44.74 ± 1.31 μm, 44.84 ± 1.92 μm, and 45.82 ± 3.29 μm respectively. (B1 to B3) Retinas of sham-treated rats at 4 weeks, 8 weeks, and 12 weeks. The average ONL thicknesses were 7.72 ± 1.01 μm, 6.71 ± 0.52 μm, 4.22 ± 0.73 μm respectively. (C1 to C3) Retinas of transplanted rats at 4 weeks, 8 weeks, and 12 weeks. The average ONL thicknesses were 28.60 ± 1.84 μm, 23.32 ± 0.84 μm, 19.82 ± 1.18 μm respectively. (**D**) Statistical analysis of ONL thicknesses in three groups at corresponding time. Data from at least three independent experiments are represented as the mean ± SD. GCL, ganglion cells layer; INL, inner nuclear layer; ONL, outer nuclear layer; SRS, subretinal space. ***P < 0.001 compared with the wild-type group; ^ooo^*P* < 0.001 compared with the sham-treated group. Cell nuclei are shown in blue; transplanted cells are shown in red. Scale bars = 100 μm.

The transplanted cells integrated into the retina and efficiently preserved the visual function of ONL (Fig. 6). The average ONL thickness of the transplanted group was 28.60 ± 1.84 μm at the 4th week, while the average ONL thickness of the sham-treated group was 7.72 ± 1.01 μm. At the 8th week, the average ONL thickness of the transplanted group was 23.32 ± 0.84 μm, while the average ONL thickness of the sham-treated group was 6.71 ± 0.52 μm. At the 12th week, the average ONL thickness of the transplanted group was 19.82 ± 1.18 μm, while the average ONL thickness of the sham-treated group was 4.22 ± 0.73 μm. Meanwhile, the ONL thickness of wild type Long-Even rats, which had normal retinal structures, were 44.74 ± 1.31 μm, 44.84 ± 1.92 μm, and 45.82 ± 3.29 μm respectively. The differences were statistically significant among the transplanted group, the sham-treated group, and the wild-type group in terms of ONL thickness (*P* < 0.001) (Fig. 6D).

### Subretinal Transplantation of RPCs Improved the ERGs of RCS Rats

**Figure 7:**
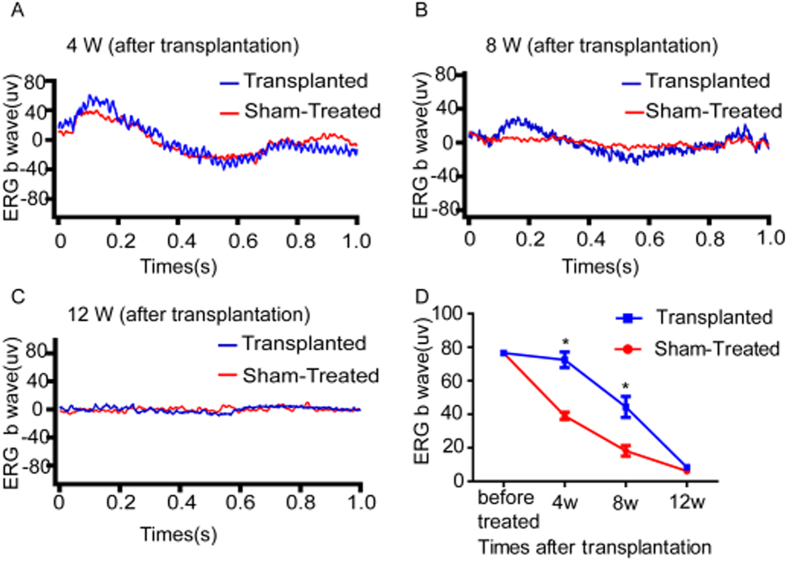
Waveforms of ERG at 4 (**A**), 8 (**B**), and 12 (**C**) weeks after transplantation. (**A** to **C**) The transplanted group did better on oscillography than the sham-treated group at 4 weeks, 8 weeks, and 12 weeks post-transplantation. (**D**) Statistical analysis of average b-wave amplitude of eyes in the transplanted group as compared with those in the sham-treated group 4, 8, and 12-weeks after transplantation. Data from at least three independent experiments are represented as the mean ± SD. *P < 0.05 compared with the sham-treated group.

In order to measure the electrical function of the host retina, bright-flash ERG responses were collected at the 4th, 8th, and 12th weeks post subretinal transplantation (Fig. 7A–C). Compared with the sham-treated group, the transplanted group had better oscillography. The average b-wave amplitude of the transplanted group was significant larger than that of the sham-treated group respectively at the 4th and the 8th week post transplantation (*P* < 0.05) (Fig. 7D). However, at the 12th week, there were no significant differences between the two groups in terms of the average b-wave amplitude (*P* > 0.05) (Fig. 7D). In summary, the partially preservation of the b-wave response reflected an improvement in the retinal function of the transplanted group as compared with the sham-treated group.

### No Tumor was found after the hESC-Derived RPCs Were Injected Into Immunodeficient Mice

**Figure 8:**
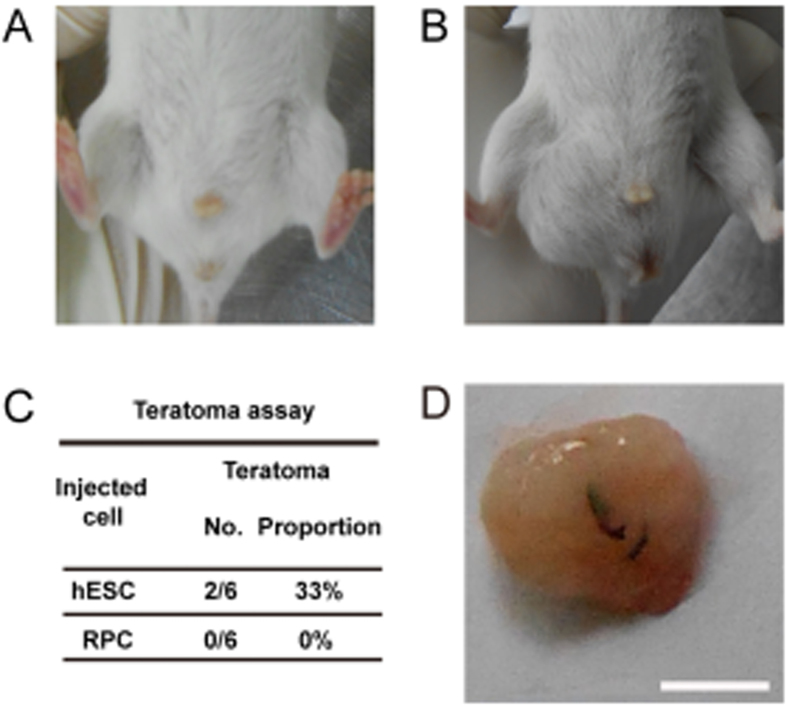
Teratoma assay of hESC-derived RPCs in SCID mice. (**A**) Teratomas were not observed in the hESC-derived RPC group. (**B**) Teratomas formation were detected in 2 of 6 SCID mice in the hESC group. (**C**) The proportion of hESC-derived RPCs and hESCs in the teratoma assay. (**D**) A teratoma derived from hESCs group. Scale bar = 0.5 cm.

To analyze the safety of transplantation, the hESC-derived RPCs were injected into the groins of 6 severe combined immune deficiency (SCID) mice; Meanwhile, the hESCs were injected into another 6 SCID mice as the positive controls. 8 weeks post injections, no gross inflammatory reaction was observed in any of the animals; no teratoma formed in the hESC-derived RPCs treated group. Nevertheless, the teratomas were found in the hESCs treated group 8 weeks after injection (Fig. 8). Histological analysis demonstrated that the formed teratomas were derived from all three germ layers. Epidermal tissue, neural tissues, cartilage, erythrocyte, muscle and intestinal epithelia, which were defined as ectoderm, mesoderm and endoderm were all identified histologically in the hESC-derived teratoma (Fig. S4).

## Discussion

Our results suggest that the hESCs can differentiate into retinal progenitor-like cells, which express the cell markers of RPCs, such as Pax6, Sox2, Rax, and Nestin. These retinal progenitor-like cells can further differentiate into the PPCs. After being transplanted into the subretinal spaces of the 21-day-old RCS rats, the hESC-derived RPCs can delay the ONL degeneration for at least 12 weeks and partially preserve the visual function for 8 weeks without any adverse effects.

Over the last few years, several studies have reported that the hESCs could differentiate into neural-like cells and photoreceptor-like cells via various inducing methods^17^,^18^,^19^. Lamba and colleagues^18^ showed that the hESCs could differentiate into RPCs when treated with mouse noggin, human recombinant Dkk-1, human recombinant IGF-1, and human recombinant bFGF. In our study, we added T3 and taurine on the 10th day of differentiation. We found that the efficiency of Crx^+^ cells derived from hESCs could reach 49.30% on the 40th day, which was much higher than that reported by Lamba and colleagues (12%)^18^. Osakada *et al*.^20^ reported that 25.4 ± 2.9% of colonies were Rax positive and 79.2 ± 5.1% of colonies were Pax6 positive on the 35th day. In our study, 66.60 ± 6.98% and 45.63 ± 2.94% of cells expressed Rax and Pax6 protein between the 10th and the 20th day. Sox2 plays an important role in maintaining the pluripotency of hESCs^21^. Interestingly, the expression of Sox2 was almost no detected in our study. This might be ascribed to the recognition of the EBs (AggreWell 400 plate incubated for 24 hours) on the 0th day. Therefore, cells on the 0th day of differentiation were not hESCs. Sox2 is also an important transcriptional factor in the retinal progenitor cells^22^. With the differentiation went on, we detected higher expression of Sox2. In the report of Osakada and colleagues, the photoreceptor progenitor marker Crx was detected on the 100th day^20^. Yanai and colleagues^16^ optimized the differentiation protocols to improve efficiency and reported that the Crx^+^ cell yield rate could reach 77%. They used the size-controlled embryonic body (EB), negative cell selection, and adding the T3 together with taurine to the differentiate culture at the appropriate time points. These studies suggest that various methods can be adopted to induce the differentiation of PPCs from hESCs. However, the Otx2-positive cell ratio was 65.40 ± 5.57% on the 20th day of differentiation, and the Crx-positive cell ratio derived from hESCs was 49.30 ± 5.10% on the 40th day of differentiation in our study. The size-controlled EB was among the most important factors. In our study, EB were manufactured using AggreWell plates to harvest the size-controlled clones with higher differentiate efficiency of RPCs. Lamba and other researchers^18^ treated the undifferentiated hESCs colonies with type IV collagenase to produce cell clumps, which were used to form inhomogeneous size EBs in a 6-well ultra-low attachment plate. In addition, the hanging-drop, round-bottomed 96-well plates, bacterial-grade dishes, 1.5 mL conical tubes, a spinner flask, and slow-turning lateral vessels collectively contributed to the formation of EBs^23^. However, these methods are disadvantageous for the EB heterogeneity. It has been proven that the heterogeneity of hESC colonies and aggregate size can directly affect the production of appropriate subsets conditions for the differentiation of specific cell types^24^. Yanai^13^ proved that a 200- and 10,000-cell EB had a lower efficiency of Crx^+^ cells differentiation. Meanwhile, by using a 1000-cell EB, the authors could harvest 77% PPCs of total cells from hESCs. Therefore, we used the AggreWell plates to form a 1000-cell EB in the present study.

In an previous study, Lamba *et al*.^25^ showed a 10% yield of Crx^+^ cells from total cells after 3 weeks of differentiation. Osakada *et al*.^26^, using a 2-dimensional method, reported a 19% yield of Crx^+^ cells from total cells after 170 days of differentiation. Nistor^27^ and Nakano^28^, used a 3-dimensional tissue construct to examine the expression levels of proteins on the 34th day of differentiation. Using an optic cup structure and the Notch inhibitor DAPT, an yield of 40–78% Crx^+^ cells from total cells was achieved^28^. In our study, however, a yield of 65.40 ± 5.57% Otx2^+^ cells from total cells was achieved on the 20th day and the rate of Crx^+^ cells from hESCs was 49.30 ± 5.10% on the 40th day of differentiation. These results were consistent with another study conducted by Yanai and colleagues^13^.

The utilization of PPCs or RPCs to treat retinal degeneration has been shown to be effective^29^,^30^. In our study, the mixed PPCs and RPCs were used; our aim was to prove that the RPCs derived from hESCs had therapeutic effects on an animal model of retinal degeneration. Before transplantation, we excluded the SSEA-4^+^ cells to minimize the occurrence of teratomas. It has demonstrated that engraftment of transplanted cells into the subretinal space of degenerative retina had neuroprotective effect^31^. We transplanted the hESC-derived RPCs into the subretinal spaces of the RCS rats and examined the structure of the host retinas by histological analysis. The ONL thickness of the transplanted RCS rat was significantly different from that of the sham-treated animal. However, The ONL thickness of the transplanted RCS rat was substantially smaller than that of the wild type rat, indicating that the protection provided by the RPCs was limited and the transplanted cell number declined over time. Taken together, these findings demonstrate that the hESC-derived RPCs could play a protective role in the host retinas, although it is difficult to maintain this improvement over time.

Retinal cells derived from hESCs have been shown to restore the light responses in animals with retinal degeneration^25^. Gonzalez-Cordero and his colleagues showed that the reliable electroretinographic responses in mice could be achieved only if at least 150, 000 functioning rods were rescued^14^. In our study, the rats that received cell implants had more positive responses than the sham-treated rats 4 weeks post transplantation. The b-wave amplitudes between the 2 groups were significantly different between the 4th and the 8th week post transplantation. But there was no significant difference between the 2 groups at 12 weeks post-transplantation. This may be attributed to the shortage of functional photoreceptors at 12 weeks post- transplantation as the ONL thickness progressively declined. These findings are consistent with another study conducted by our team^32^. Therefore, these results suggest that the hESC-derived RPCs are effective in preserving visual function, although the protective effects are limited to 8 weeks post-transplantation. However, the RPCs transplanted into the subretinal spaces of RCS rats at least delay the progression of retinal degeneration. It has been reported that transplanted stem cells can distributed themselves throughout multiple neuroretinal layers^33^. In our study, however, the transplanted RPCs migrated mainly to the ONL and subretinal space.

In the end, we analyzed the safety of hESC-derived RPCs by teratoma assay. There was no evident tumor formation 8 weeks after the transplantation of hESC-derived RPCs, indicating that the injection of hESC-derived RPCs is a relatively safe strategy.

In summary, our study proves that the transplantation of RPCs, which are derived from hESCs within a relatively short period of time, can improve the retinal structure and partially preserve the visual function of the degenerative retina.

## Materials and Methods

### Culture of hESCs

The H1 cells (WA01) (WiCell Research Institute, Madison, WI)^34^ were a gift from Professor Huang Yue of the Chinese Academy of Medical Sciences & Peking Union Medical College. They were grown in the precoated plates with Matrigel (BD Bioscience, Franklin Lakes, NJ) in mTeSR1 (STEMCELL Technologies, Vancouver, Canada) at 37 °C in a humidified atmosphere containing 5% CO_2_. The medium was changed daily and the cells were passaged with accutase (STEMCELL Technologies) every 4 to 7 days.

### Differentiation

At time to passage we aspirated the maintenance medium from the plate (1 well of 6-well dishes), and rinsed the cells once with 2 mL of DMEM/F12 (Hyclone, South Logan, UT). After discarding DMEM/F12, 1 mL of accutase was added to cover the cells, which were incubated for 3 to 5 minutes at 37 °C in a humidified atmosphere containing 5% CO_2_. The plate was inspected microscopically to ensure that the cells were peeling off the plate. The cell suspension culture was gently pipetted several times to dissociate the cell clumps. The accutase was diluted with DMEM/F12. Then the cell suspension was centrifuged at 200 g for 3 minutes, resuspended in appropriate EB formation medium mTeSR1 (the cell concentration was approximately 1.2 × 10^6^ cells/mL) and was supplemented with 10 μM Y27632 (STEMCELL Technologies). Then 1 mL of cell resuspension solution was added to 1 well of the AggreWell 400 plate (STEMCELL Technologies), which was centrifuged for 3 minutes at 100 g at room temperature to capture the cells into the microwells of the AggreWell 400 plate. After incubating the cells for 24 hours at 37 °C in a humidified atmosphere containing 5% CO_2_, most of the cells formed the EBs located in the micro-wells. Then the EBs were harvested from micro-wells by firmly pipetting medium in the well up and down several times with Pasteur pipette to dislodge most of the EB from the microwells (We recognized these cells on the 0th day). Then the EB were placed on an ultra-low attachment plate for 3 days in an EB suspension solution containing DMEM/F12, knockout serum replacement, B27 and N2 supplements (Life Technologies, Waltham, MA), 1 ng/mL recombinant human DKK1, 1 ng/mL mouse noggin, and 5 ng/mL recombinant human insulin-like growth factor-1 (IGF-1) (R&D Systems, Minneapolis, MN). On the fourth day, all EBs were collected and resuspended with differentiation medium and then distributed on 6-well dishes or coverslips precoated with matrigel. The differentiation medium, which was called a retinal differentiation (R&D Systems) culture, contained DMEM/F12, B27 and N2 supplements (Life Technologies), 10 ng/mL recombinant human DKK1, 10 ng/mL mouse noggin, 10 ng/mL recombinant human IGF-1, and 5 ng/mL recombinant human basic fibroblast growth factor (bFGF). (Life Technologies). After day 10, triiodothyronine (T3, Sigma, St Louis, MO) and taurine (Sigma) were added to the differentiation medium. (Fig. S3)

### Flow Cytometry

To collect the cells, we washed them with cold PBS, and added 1 mL accutase for one well of six-well dishes. The cells were incubated for 5 to 30 minutes at 37 °C until they peeled off the plate. Pipetted the cells up and down several times, diluted the accutase with PBS and transferred them into a 15-mL tube. The tube was spun at 300 g for 3 minutes at room temperature. They were then washed twice to remove any residual growth factors or accutase and then counted and distributed into several 1.5-mL Eppendorf tubes.

For cell surface marker, stage-specific embryonic antigen-4 (SSEA-4), one sample had 100,000 cells used. We added 100 μL of staining buffer and 2 μL of Fc block per tube, vortexed this softly, and blocked the cells for 15 minutes at room temperature. We then added conjugated antibody (BD Bioscience) and vortexed again. The cells were incubated for 30 minutes at 4 °C in the dark. And then we removed any unbound antibody by washing the cells in staining buffer. The suspended cells were centrifuged at 300 g for 5 minutes; this was repeated once to discard the redundant antibody. Finally the cells were resuspended in 500 μL of staining buffer supplemented with 7-AAD for flow cytometric analysis with the fluorescence-activated cell sorter (FACS) Aria II (BD Bioscience, Franklin Lakes, NJ).

For intracellular labeling, 500,000 cells per sample were needed. The cells were fixed with 250 μL of 4% paraformaldehyde per sample for 15 minutes at 4 °C and centrifuged at 300 g for 5 minutes. Then the supernant was discarded and the cells permeabilized with saponin (Sigma) for 15 minutes at room temperature. Thereafter the cells were incubated for 30 minutes at 4 °C with the following primary antibodies: mouse anti-Ki67 (1:200) (BD Bioscience), rabbit anti-Pax6 (1:200) (Santa Cruz, Dallas, TX), rabbit anti-Sox2 (1:200) (Abcam, Cambridge, MA), rabbit anti-Rax (1:200) (Abcam), rabbit anti-Nestin (1:200), rabbit anti-Otx2 (1:200), rabbit anti-Crx (1:200), or rabbit anti-Recoverin (1:200) (Millipore, Billerica, USA). Each sample was washed twice with 1 mL saponin to remove the unbound primary antibody. Thereafter the cells were resuspended in 100 μL FITC goat anti-rabbit secondary antibody (1:200, BD Bioscience). The cells were incubated for 30 minutes at room temperature, following by 2 washings with saponin. They were then resuspended with 300 μL of 2% FBS and tested using FACS Aria II. Data were analyzed with CellQuest Pro Software (BD Bioscience).

### Cell Cycle

The cell-harvesting procedure described above yielded consistent results. The cells were fixed after cooling in 70% ethanol solution overnight and then stained using a PI/RNase reagent kit. DNA content was determined using a BD Accuri C6 Flow Cytometer, and the data were analyzed using Mod Fit 2.0 software (BD Bioscience). At least 20,000 cells in each sample were analyzed.

### Immunofluorescence

The cells were gently rinsed twice with PBS and then fixed with 4% paraformaldehyde at room temperature for 15 minutes, followed by triple rinsing with PBS. The cells were then permeabilized with 0.1% triton-X for 10 minutes at room temperature. Primary antibodies—mouse anti-Ki67 (1:200; BD), rabbit anti-Pax6 (1:200, Santa Cruz), rabbit anti-Sox2 (1:200, Abcam), rabbit anti-Rax (1:200, Abcam) or rabbit anti-Nestin (1:200, Santa Cruz) were added to incubate for overnight. Then primary antibodies were stained with secondary antibodies goat antirabbit conjugated to FITC (1:100, CWBIO, Beijing, China). Cell nuclei were counterstained with DAPI (1:800, Beyotime, Shanghai, China). Cells were counted and imaged in optical slice images with a fluorescence microscope (Olympus Microsystem, Japan).

### Fluorescence-Activated Cell Sorting (FACS)

On day 20, the differentiated cells were digested with accutase for 10 to 20 minutes. Then 3 mL of wash buffer (Biolegend, San Diego, CA) was added, followed by centrifugation at 300 g for 3 minutes at 4 °C. Thereafter the collected cells were resuspended with Stain Buffer (Biolegend) and 2 μL of Fc block (Biolegend) was added, followed by incubation for 15 minutes at 4 °C. At this point fluorochrome antibody conjugates were added and the cells were incubated for 30 minutes at 4 °C while shaking, followed by washing and centrifugation at 300 g for 5 minutes at 4 °C. After the supernatant was removed, 300 μL of PBS (Hyclone) was added to resuspend the cells. The suspension was then transferred to a standard flow cytometry tube before being analyzed using FACS Aria II (BD Bioscience).

### Western Blot Analysis

Western blot analysis was performed as previously described^35^. Total protein was isolated from the differentiated cells using Total Protein Extraction Lysis Buffer (Bioworld, Louis Park, USA) following manufacturer’s instructions. BSA was used as a standard to measure protein concentration of each sample using the Bio-Rad Protein Assay (Bio-Rad, California, USA). Then eluted proteins were analyzed by SDS-PAGE/Western blot. Primary antibodies were incubated at 4 °C overnight (rabbit anti- Pax6 [Abcam] 1:500; rabbit anti-Sox2 [Abcam] 1:500; rabbit anti-Rax [Abcam] 1:500; rabbit anti-Nestin [Abcam] 1:500; rabbit anti-Crx [Santa Cruz] 1:200; rabbit antirecoverin [Millipore] 1:500; rabbit anti-GAPDH [Abcam] 1:2000). Then secondary antibody (goat anti-rabbit IgG H&L HRP, [Abcam] 1:2000) was used to conjugate with primary antibody for 1 hour at 37 °C with shaking. Finally, protein bands were detected using Fluorchen R (ProteinSimple, San Jose, California, USA) and analyzed with Image J software.

### Real-Time Polymerase Chain Reaction (PCR) Analysis

Gene encoding signal transduction proteins were analyzed using real-time reverse transcription PCR. Total RNA was isolated from differentiated cells using TRIzol reagent (Invitrogen, California, USA), then reverse transcription was performed using HiScript Q RT SuperMix (Vazyme, Nanjing, China). Real-time PCR analysis was performed using the SYBR Green Master Mix (Vazyme) and ABI 7900HT system following manufacturer’s instructions. The real-time PCR program was performed as follows: 40 cycles of denaturation at 95 °C for 10 seconds, annealing at for 30 seconds, elongation at 56–60 °C for 30 sec. Nonspecific amplicons were appeared determined by the melting curves. The internal control was *GAPDH. Pax6, Sox2, Rax, Nestin, Otx2, Crx* and *Recoverin* were analyzed. The primer sequences of the genes are listed in Table S1.

### Animal Feeding

Rats were fed and housed under a 12 hour light-dark cycle. The animal protocol was approved by the Institutional Animal Care and Use Committee of the Third Military Medical University in accordance with the National Institutes of Health guidelines for the care and use of laboratory animals, and with the Use of Animals in Ophthalmic and Visual Research (ARVO) statement. Cyclosporine A (210 mg/L) was added in the drinking water of rats from the first day prior transplantation until they were euthanized^36^.

### Subretinal Transplantation

Differentiated cells were harvested according to the previous method on day 20. After removing the SSEA-4-positive cells by FACS, cells were stained with CM-Dil (Molecular Probes) for 5 minutes at 37 °C in a humidified atmosphere containing 5% CO_2_ and then incubated for an additional 15 minutes at 4 °C. After that, they were with PBS twice and resuspended in fresh medium.

Rats with congenital disease, such as microphthalmia and congenital cataract, were excluded from our study. The RCS rats without microphthalmia or congenital cataract were randomly divided into 2 groups: the transplanted group (n = 9) and the sham-treated group (n = 9).

Twenty-one-day-old RCS rats were anesthetized with 4% chloral hydrate (0.8 mL/100 g of body weight) and pupils dilated with drops of 1% tropicamide. A syringe (Hamilton) with a blunt, 32-gauge needle was inserted tangentially through the sclera and placed into the subretinal space using a surgical microscope. A suspension solution containing approximately 6 × 10^5^ differentiated cells was injected into the subretinal space in a 3-μL solution (transplanted group, right eyes), or 3-μL HBSS were injected into the subretinal space (sham-treated group, right eyes). Cyclosporine A (210 mg/L) in drinking water was added to suppress rejection throughout the entire post-transplantation period^36^.

Long-Even rats, a normal rat model, were used in the research as a wild-type group.

### Electroretinography

The b-wave amplitude of dark-adapted full field ERG was a good indicator to predict the degree of visual field in the eye^37^. The dark-adapted ERG response was recorded as previously described^38^. In brief, mice were dark-adapted at least 12 hours, then anesthetized as described for the surgical procedure above. Eyes were dilated with 1% tropicamide. A contact electrode was placed on each cornea, a ground needle electrode under tail skin, and reference electrodes under the scalp near the ears. We used retiport 32 4.3.8 software of the Roland Electrophysiological Systems (Brandenburg, Germany) to acquire amplification (0.1–300 Hz bandpass without notch filtering), and stimulated presentation and data acquisition. To improve the signal-to-noise ratio, we repeated this at least 3 times to record single flash presentations.

### Histological Analysis

After recording ERG, some mice were anesthetized as the above process. Then the mice were perfused with 0.9% NaCl followed by 4% paraformaldehyde. Eyeballs were dissected out and fixed in 4% paraformaldehyde for 30 minutes, then the cornea and lens were discarded, after that, the eyes were fixed in 4% paraformaldehyde for another 90 minutes. Then, eyes were infiltrated with 30% sucrose overnight and then embedded in an optimal cutting temperature compound (OCT). Sections were cut on a cryostat (Leica CM190, Leica Microsystems, Wetzlar, Germany), and were restored at −20 °C.

Sections were prewarmed to room temperature for 20 minutes and washed twice before being stained. Then cell nuclei were counterstained with DAPI. The cells were counted and imaged in optical slice images with fluorescence microscopy (Leica Microsystems, Wetzlar, Germany).

For cell counting, we placed slices in the order of section and selected a slice in the middle for staining. The number of surviving cells was counted in a microscopic field at 400x magnification in the middle of one section.

### Teratoma Assay

RPCs (1 × 10^7^/100 μL) derived from hESCs were injected into the groins of 6 SCID mice; hESCs (1 × 10^7^/100 μL) were injected into 6 SCID mice as positive control. These animals were observed for 8 weeks to detect possible tumor formation. Teratomas were collected, embedded in paraffin and examined histologically after staining with hematoxylin-eosin (HE).

### Statistical Analysis

Data from at least 3 independent experiments were all represented as mean ± standard deviation (SD). Statistical analyses were carried out using the SPSS 17.0 software using either Student’s 2-tailed t-test or 1-way analysis of variance. *P* < 0.05 was defined as statistically significant.

## Additional Information

**How to cite this article**: Shao, J. *et al*. Experimental Study of the Biological Properties of Human Embryonic Stem Cell–Derived Retinal Progenitor Cells. *Sci. Rep.* **7**, 42363; doi: 10.1038/srep42363 (2017).

**Publisher's note:** Springer Nature remains neutral with regard to jurisdictional claims in published maps and institutional affiliations.

## Supplementary Material

Supplementary Information
