# Supplementary material for: Experimental Study of the Biological Properties of Human Embryonic Stem Cell–Derived Retinal Progenitor Cells
Source: Sci Rep. 2017 Feb 13;7:42363. doi: 10.1038/srep42363 (PMC5304228; doi:10.1038/srep42363)
Supplement: Supplementary Information [file srep42363-s1.pdf]

Experimental Study of the Biological Properties of Human Embryonic Stem Cell–Derived Retinal Progenitor Cells

Jingzhi Shao, [1]Peng-Yi Zhou, [2]Guang-Hua Peng[a,3]

[1]Department of Ophthalmology, The First Affiliated Hospital of Zhengzhou University, Zhengzhou, Henan 450000, China. shaojingzhi@gs.zzu.edu.cn

[2]Department of Ophthalmology, The First Affiliated Hospital of Zhengzhou University, Zhengzhou, Henan 450003, China. zhoupengyi@zzu.edu.cn

[3]Department of Ophthalmology, The First Affiliated Hospital of Zhengzhou University, Zhengzhou 450000, China; Department of Ophthalmology, General Hospital of Chinese People's Liberation Army, Beijing 100853, China. ghp@zzu.edu.cn

[a]Corresponding author: Guang-Hua Peng, email: ghp@zzu.edu.cn

[Supplemental materials]

Table S1. The Primer Sequences of Specific Genes in Differentiated Cells Used for Real-Time PCR Analysis

Gene	Primer sequences (5'-3')	Product length (bp)
Pax6	CCGTGGCTCGGCCTCATTTC(F)<br>ACCGCTCCTCACTGGCCCATTA(R)	107
Sox2	CGCCCCCAGCAGACTTCA CA(F)<br>CTCCTCTTTTGCACCCCTCCCATTT(R)	170
Rax	AACCGCACGACTTTCACCACG(F)<br>TGCAGCTTCATGGAGGACACTTC(R)	200
Nestin	GAATCACTGAAGTCTGCGGGAC(F)<br>TCCAGGAGTCTGAATGTCTCTTGG(R)	176
Otx2	ACCCGGTAGTGTGTCCCGCT(F)<br>TCGCCGCTCTCTTCCAGGGT(R)	208
Crx	CCTTCTGACAGCTCGGTGTT(F)<br>TGGTGTACTTCAGCGGTCAC(R)	149
Recoverin	GGAAAAGCGAGCCGAGAAGA(F)<br>CCTGGGGTGGATGTGTGTGT(R)	282
GAPDH	AGCCTCCCGCTTCGCTCTCT(F)<br>CCAGGCGCCCAATACGACCA(R)	141

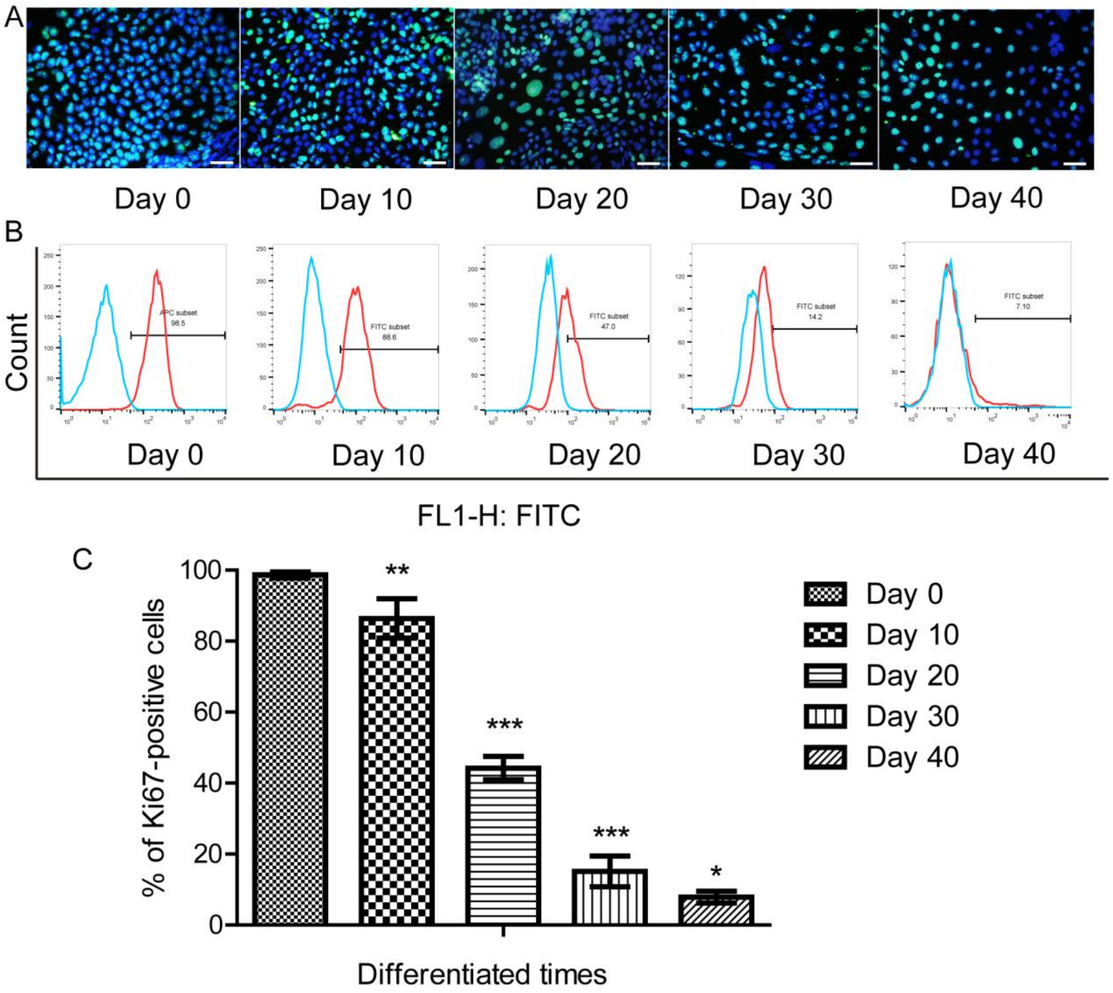

Figure. S1 The expression of protein Ki67 on day 0, 10, 20, 30 and 40 of differentiation. (A) The expression of protein Ki67 decreased over time evaluated by immunofluorescence. (B) Staining of Ki67 was evaluated by using flow cytometry. Blur line, isotype. (C) Statistical analysis of expression of Ki67. Cell nuclei are shown in 6-diamidino-2-phenylindole (DAPI), blue. Data from at least three independent experiments are represented as the mean ± SD. Scle bar = 100 μm. *P<0.05, **P<0.01, ***P<0.001, versus with data of previous time point.

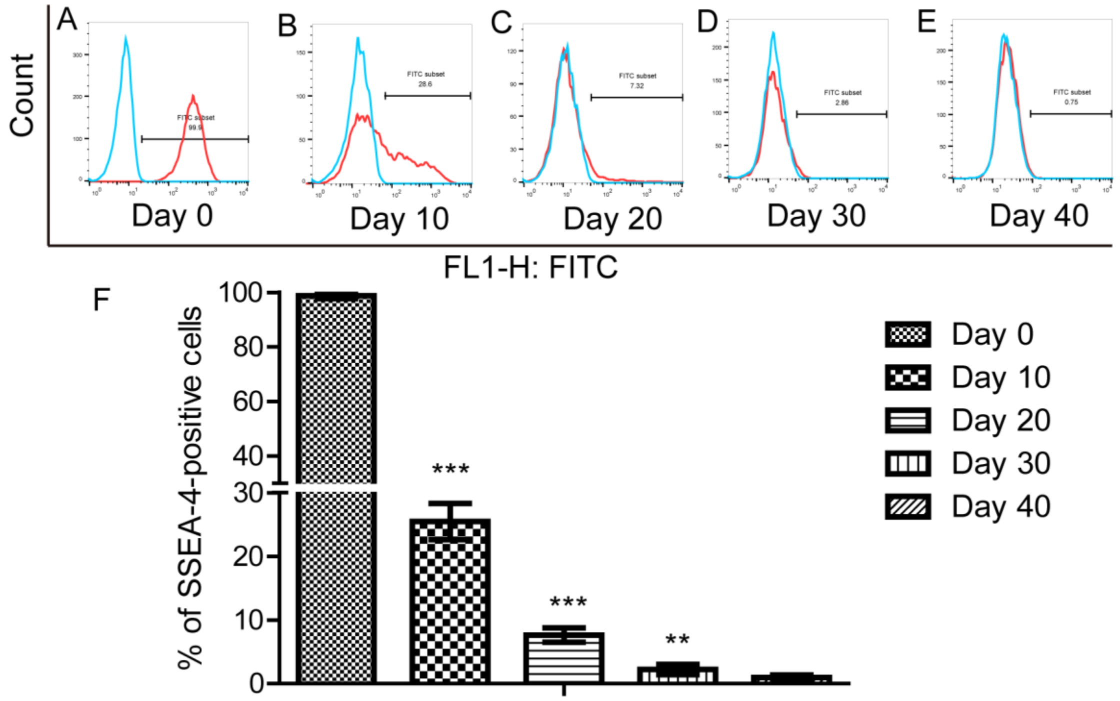

Figure. S2 The expression of protein SSEA-4 at day 0, 10, 20, 30 and 40 of differentiation. (A) The expression of protein SSEA-4 decreased over time evaluated by flow cytometry. (B) Statistical analysis of expression of SSEA-4. Data from at least three independent experiments are represented as the mean ± SD. **P <0.01, ***P <0.001, versus with data of previous time point.

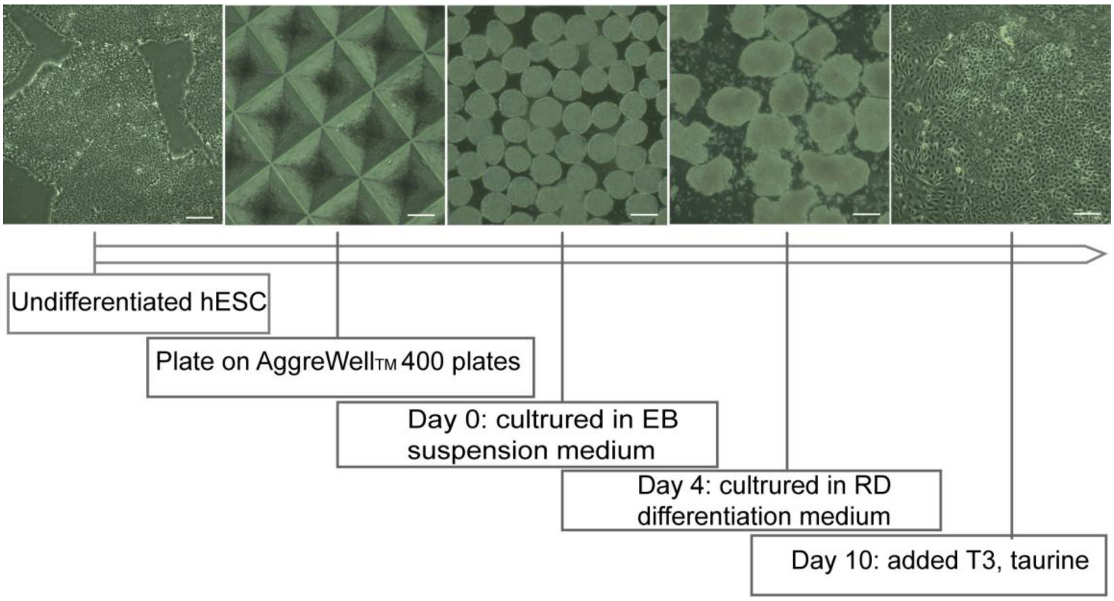

Figure. S3 Flow chart of hESC differentiation.

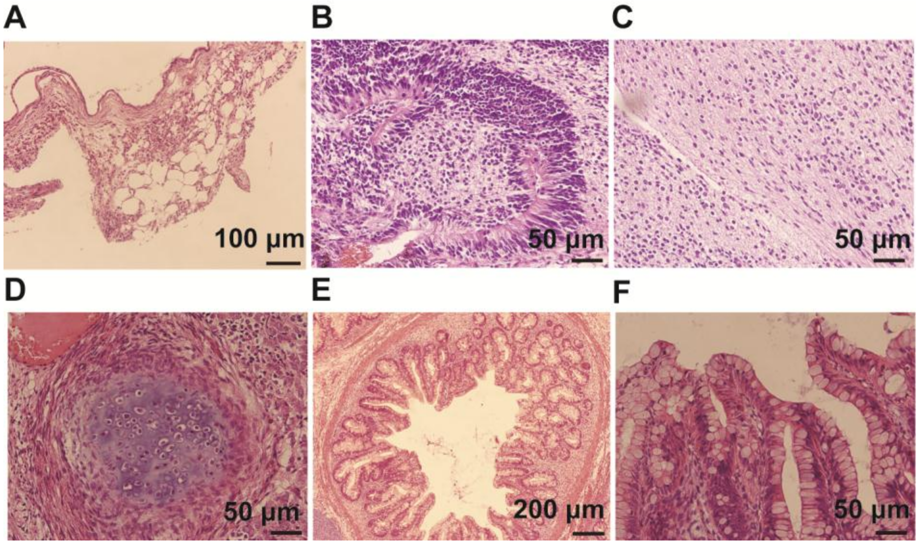

Figure. S4 Hematoxylin-eosin assay of teratoma. (A) Epithelium that was defined as ectoderm. (B and C) Neural tissues which were defined as ectoderm. (D) Cartilage, fibrous tissues and erythrocyte which were defined as mesoderm. (E) Circular muscle and gut-like epithelium with mucous-containing cells which were defined as endoderm. (F) Gut-like epithelium, which was defined as endoderm.
